# Serum neurofilament light chain as a severity marker for spinocerebellar ataxia

**DOI:** 10.1038/s41598-021-92855-z

**Published:** 2021-06-29

**Authors:** Hye-Rim Shin, Jangsup Moon, Woo-Jin Lee, Han Sang Lee, Eun Young Kim, Seoyi Shin, Soon-Tae Lee, Keun-Hwa Jung, Kyung-Il Park, Ki-Young Jung, Sang Kun Lee, Kon Chu

**Affiliations:** 1 Department of Neurology, Dankook University Hospital, Cheonan, Chungnam South Korea; 2 Department of Neurology, Seoul National University Hospital, Seoul National University College of Medicine, 101 Daehak-ro, Jongno-gu, Seoul, 110-744 South Korea; 3 Laboratory for Neurotherapeutics, Center for Medical Innovations, Biomedical Research Institute, Seoul National University Hospital, Seoul, South Korea; 4 Department of Genomic Medicine, Seoul National University Hospital, Seoul, South Korea; 5 Center for Hospital Medicine, Seoul National University Hospital, Seoul, South Korea; 6 Department of Neurology, Chungnam National University Sejong Hospital, Sejong, South Korea; 7 Department of Neurology, Seoul National University Healthcare System Gangnam Center, Seoul, South Korea

**Keywords:** Neuroscience, Medical research

## Abstract

Since the serum neurofilament light (NfL) chain is known as a promising biomarker in neurodegenerative diseases, we aimed to evaluate serum NfL as a biomarker indicating neuronal damage in autosomal-dominant (AD) spinocerebellar ataxia (SCA). We reviewed patients diagnosed with AD SCA in the outpatient clinic of Seoul National University Hospital’s (SNUH) Department of Neurology between May and August of 2019. We reviewed the demographic data, clinical characteristics, Scale for the Assessment and Rating of Ataxia (SARA) score, and brain magnetic resonance imaging (MRI) scans. The serum NfL was measured by electrochemiluminescence (ECL) immunoassay. Forty-nine patients with AD SCA were reviewed and their serum NfL level was determined. The median serum NfL level (109.5 pg/mL) was higher than control (41.1 pg/mL) (p-value < 0.001). Among the AD SCA patients, there was a positive correlation between the serum NfL level and the trinucleotide repeat number (r = 0.47, *p*-value = 0.001), disease duration (r = 0.35, *p*-value = 0.019), disease duration/age × trinucleotide repeat number (r = 0.330, *p*-value = 0.021), and SARA score (n = 33; r = 0.37, *p*-value = 0.033). This study shows that serum NfL is elevated in AD SCA patients and correlates with clinical severity.

## Introduction

Spinocerebellar ataxia (SCA) is heterogeneous group of inherited disorders with progressive ataxia, and autosomal-dominant (AD) SCA is the major subgroup^1–3^. AD SCA shares a common pathomechanism of abnormally expanded CAG trinucleotide repeats, resulting in progressive loss of cerebellar Purkinje cells^1–3^.

The disease severity and prognosis of AD SCA is highly variable^1,2^. Hence, a biomarker is needed to track disease progression^1,4^. Clinical severity scales, such as the Scale for the Assessment and Rating of Ataxia (SARA)^5^, are widely used to assess disease progression. Additionally, MRI^6^, magnetic resonance spectrography^7^, trinucleotide repeat number^1,2,4^, cytokines^8^, and CSF-tau^9^ have been studied. However, there is no broadly accepted biomarker in SCA.

Neurofilaments are a component of the axonal cytoskeleton including neurofilament light chain (NfL)^10–12^. When axonal damage occurs, neurofilaments are released into the cerebrospinal fluid (CSF) and the blood, and NfL can be detected in blood^10–12^. In previous studies, serum NfL has been demonstrated as a potential biomarker in various neurodegenerative diseases^10–14^. Also in recent studies with SCA 3 patients, the serum NfL level was significantly elevated and correlated with clinical severity^15,16^. However, no prior research has implied that serum NfL is a severity indicator, including in diverse types of AD SCA.

In this study, we aimed to determine whether serum NfL could be a biomarker indicating clinical severity in AD SCA. To demonstrate this hypothesis, we measured the serum NfL levels in various types of AD SCA patients and compared them to the controls. Next, we evaluated the correlation between serum NfL and clinical severity.

## Results

### Clinical characteristics and genetic testing

Forty-nine patients with AD SCA were enrolled (Table 1 and Supplementary Table S1). Supplementary Table S2 portrays patients’ detailed information. Among them, 31 (63.3%) were female, and the median age was 44.0 years (34.0–50.0 years). Genetic testing confirmed a diagnosis of AD SCA for all participants. The SCA types included SCA 2 (19, 38.8%), SCA 3 (13, 26.5%), SCA 6 (9, 18.4%), SCA 7 (4, 8.2%), SCA 1 (2, 4.1%), and SCA 17 (2, 4.1%). The median trinucleotide repeat number was 42.0 (38.0–65.0).

**Table 1:** Clinical characteristics of autosomal-dominant spinocerebellar ataxia patients and serum neurofilament light chain levels.

Subject characteristics	Total (n = 49)
Age—year	44.0 (34.0–50.0)
Female sex—no. (%)	31 (63.3)
**SCA type—no. (%)**	
SCA 1	2 (4.1)
SCA 2	19 (38.8)
SCA 3	13 (26.5)
SCA 6	9 (18.4)
SCA 7	4 (8.2)
SCA 17	2 (4.1)
Trinucleotide repeat number	42.0 (38.0–65.0)
Disease onset—year	38.0 (27.0–46.0)
Disease duration—year	5.0 (2.0–7.0)
SARA (n = 33)	11.0 (9.0–15.0)
Serum NfL (pg/mL)	109.5 (70.1–154.9)

Data are reported as the number (percentage), or as the median (interquartile range, IQR).

*SCA* spinocerebellar ataxia, *SARA* scale for the assessment and rating of ataxia, *NfL* neurofilament light chain.

The median age at disease onset was 38.0 years (27.0–46.0 years), and the median disease duration was 5.0 years (2.0–7.0 years). A disease severity score of 33 patients was obtained using the SARA, and the median score on the SARA was 11.0 (9.0–15.0).

Fourteen patients had received brain MRI scans. The cerebellar volume was 107.0 cm^3^ (103.2–113.2 cm^3^), and the TIV was 1407.0 cm^3^ (1325.8–1560.1 cm^3^). The median percentage of cerebellar volume within the TIV was 7.5% (7.0–8.1%).

### Serum NfL levels in AD SCA patients and controls

Among the control subjects, 28 (75.7%) were female, and the median age was 42.0 years (33.0–49.0 years). There was no significant difference in sex (*p*-value = 0.220) or age (*p*-value = 0.441) between the SCA patients and the controls.

**Figure 1:**
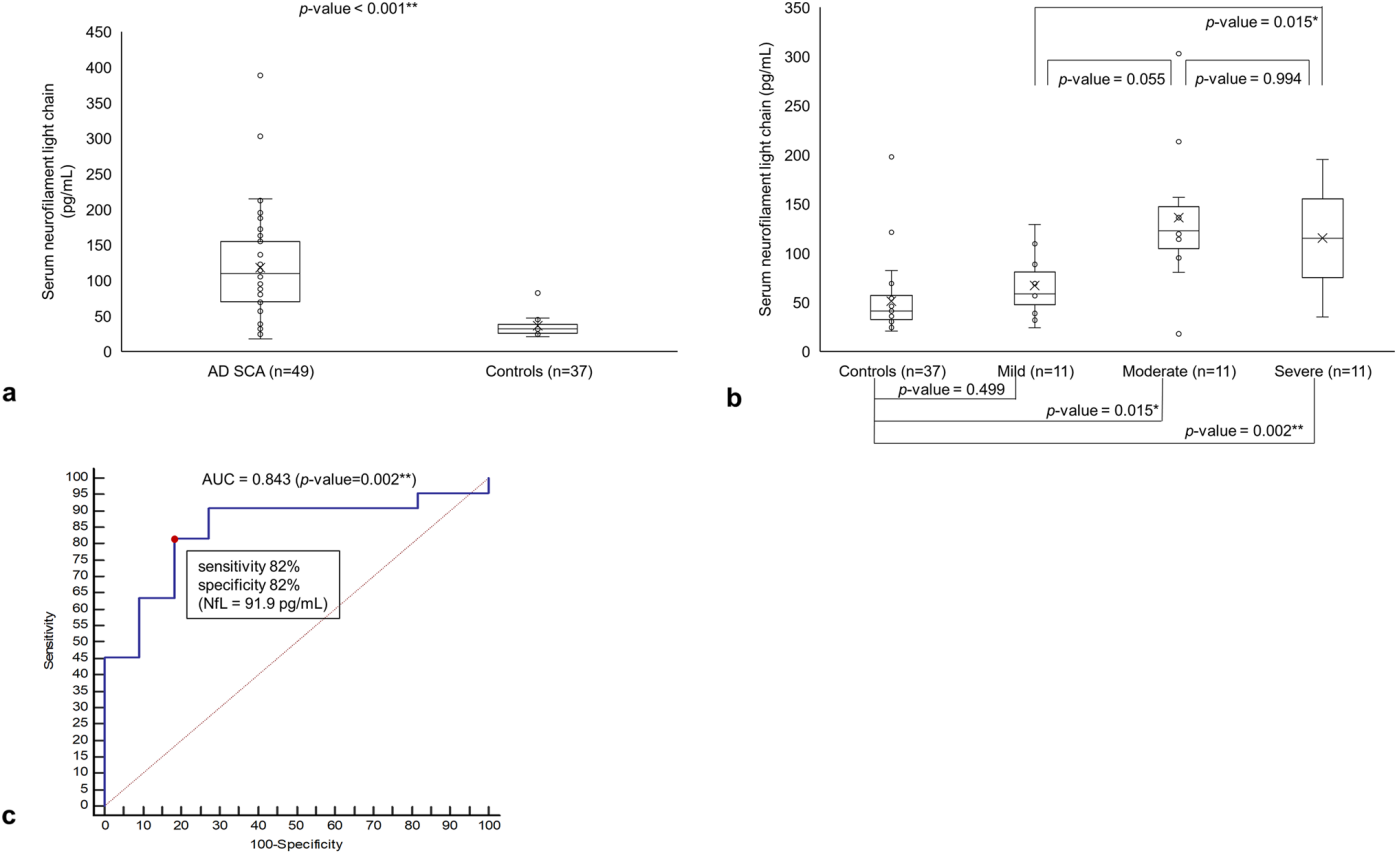
Serum neurofilament light chain in different severity groups of autosomal-dominant SCA and the controls. **(a)** Comparison of serum NfL chain level in autosomal-dominant SCA patients (n = 49) and the controls (n = 37). **(b)** Comparison of NfL levels among mild (SARA score ≤ 10), moderate (10 < SARA score ≤ 13), and severe (SARA score > 13) groups of autosomal-dominant SCA patients. **(c)** ROC curve of serum NfL level for distinguishing moderate and severe autosomal-dominant SCA patients from mild SCA patients. Area under the curve (AUC value was 0.843 (95% CI, 0.702–0.984, *p*-value = 0.002). ** is for *p*-value < 0.01, and * is for *p*-value < 0.05.

The median serum NfL level in the AD SCA patients was 109.5 pg/mL (70.1–154.9 pg/mL), which was significantly higher than that among the controls (median: 41.1 pg/mL, 32.3–57.1 pg/mL) (*p*-value < 0.001) (Fig. 1a, Table 2).

**Table 2:** Patient characteristics and serum neurofilament light chain of autosomal-dominant spinocerebellar ataxia patients and controls.

	AD SCA (n = 49)	Controls (n = 37)	p-value
Age—year (range)	44.0 (34.0–50.0)	42.0 (33.0–49.0)	0.441
Female sex—no. (%)	31 (63.3)	28 (75.7%)	0.220
Serum NfL (range) (pg/mL)	109.5 (70.1–154.9)	41.1 (32.3–57.1)	< 0.001

Data are reported as the number (percentage), or as the median (interquartile range, IQR).

*AD* autosomal dominant, *SCA* spinocerebellar ataxia, *NfL chain* neurofilament light chain.

We also compared the serum NfL levels of AD SCA patients with those of healthy control groups used in two previous studies, and we found that the serum NfL level in the AD SCA patients was much higher than that in the healthy controls (Supplementary Table S3). The first study, involving ALS, analyzed 34 healthy controls; the median serum NfL level was 10.7 pg/mL (range: 0.4–33.5 pg/mL)^14^. The other study, which examined patients with various neurodegenerative diseases, included 67 healthy controls; the median serum NfL level was 3.3 pg/mL (IQR 2.0–5.4 pg/mL) ^10^. Considering that these studies used the same method of measuring the serum NfL level as we did in our study, this suggests that the serum NfL level is consistently higher in AD SCA patients.

### Serum NfL levels in patients with different severities of AD SCA

We classified the AD SCA patients into three subgroups according to SARA score and compared the serum NfL levels between the different severity groups and the controls (Fig. 1b). In the mild (SARA score ≤ 10; n = 11), moderate (10 < SARA score ≤ 13; n = 11), and severe (SARA score > 13; n = 11) groups, the median SARA score was 8.5 (7–9), 11 (10.8–11.8), and 17 (15–27), respectively; the median serum NfL level was 58.5 pg/mL (47.7–80.9 pg/mL), 122.7 pg/mL (104.7–147.4 pg/mL), and 114.5 pg/mL (100.2–172.4 pg/mL), respectively.

The serum NfL level was significantly higher in the moderate and severe groups (*p*-value = 0.015, and *p*-value = 0.002, respectively) than in the control group, but in the mild group, it was not different (*p*-value = 0.499). Among the severity groups, the difference in the serum NfL level was significant between the mild and severe groups (*p*-value = 0.015) but not between the mild and moderate groups (*p*-value = 0.055) or between the moderate and severe groups (*p*-value = 0.994).

We also compared the serum NfL levels between the different severity groups and the controls after adjusting for age. The serum NfL level was significantly higher in the moderate and severe groups (*p*-value = 0.005, and *p*-value = 0.001, respectively) than in the control group, but the difference was not significant in the mild group (*p*-value = 0.296). However, the serum NfL level was not significantly different when comparing the severity groups to one another (mild and moderate groups: *p*-value = 0.135; mild and severe groups: *p*-value = 0.101; moderate and severe groups: *p*-value = 0.998).

The ROC curve showed that the serum NfL level was sensitive and specific for distinguishing moderate and severe AD SCA patients from the mild group. The AUC curve of the moderate and severe AD SCA groups was 0.843 (95% confidence interval [CI]: 0.702–0.984, *p*-value = 0.002, Fig. 1c). The serum NfL level, at 91.9 pg/mL, presented the highest sensitivity and specificity for distinguishing between the moderate and severe groups of SCA patients (sensitivity: 82%; specificity: 82%).

### Correlation analysis with severity markers

**Figure 2:**
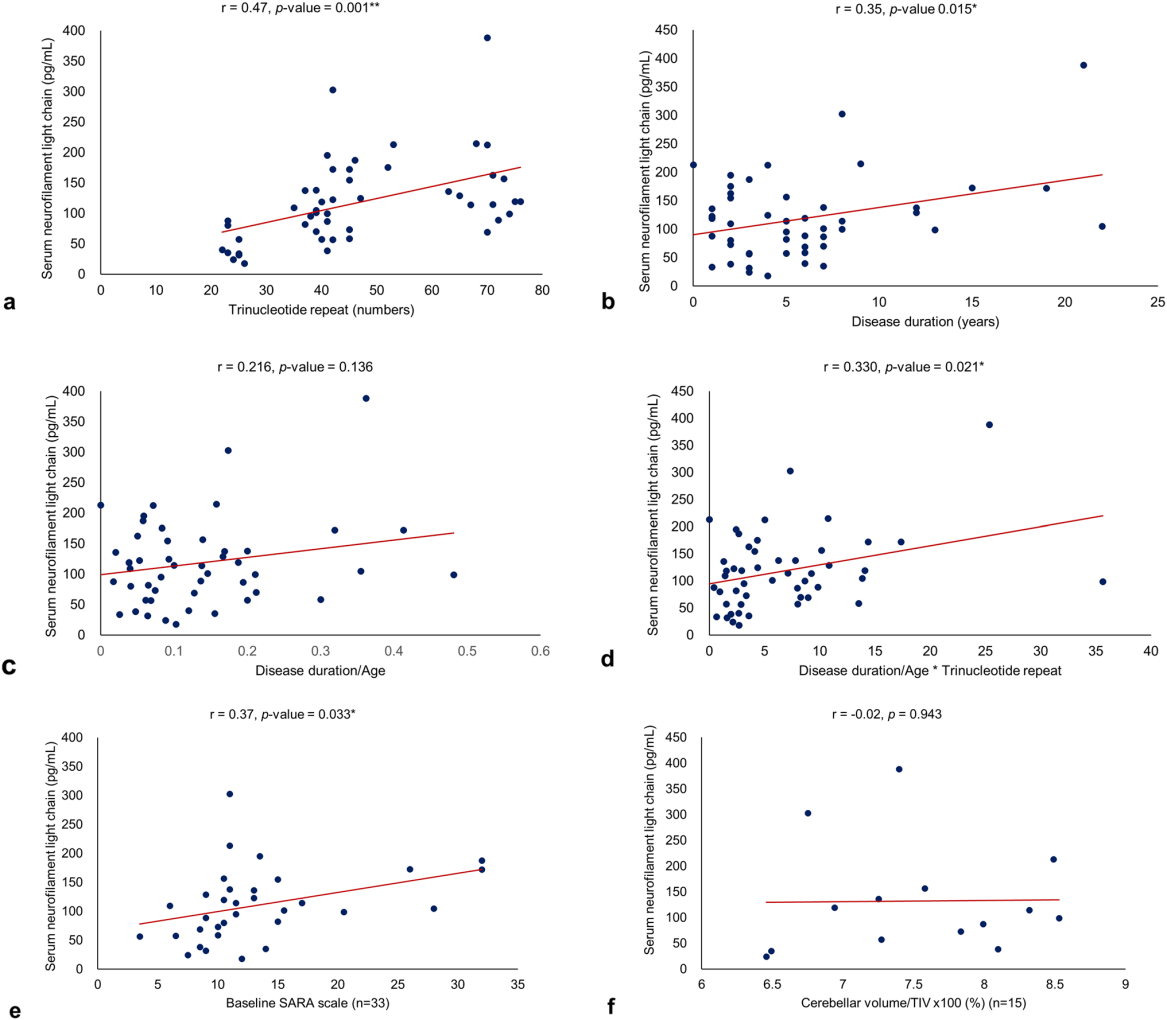
Correlation of serum neurofilament light chain with clinical severity of autosomal-dominant SCA.** (a)** The trinucleotide repeat number and serum neurofilament light chain showed a positive correlation (r = 0.47, *p*-value = 0.001). **(b)** Disease duration and the serum neurofilament light chain showed a positive correlation (r = 0.35, *p*-value = 0.015). **(c)** Disease duration and age were not significantly correlated (r = 0.22, *p*-value = 0.136). **(d)** The disease duration/age × trinucleotide repeat number showed a positive correlation with serum NfL (r = 0.33, *p*-value = 0.021). **(e)** The SARA score and serum neurofilament light chain were positively correlated (n = 33; r = 0.37, *p*-value = 0.033). **(f)** The percentage of cerebellar volume within TIV did not have a significant correlation with serum NfL (n = 14; r = 0.02, *p*-value = 0.957). ** is for *p*-value < 0.01, and * is for *p*-value < 0.05.

To evaluate the relationship between the serum NfL level and clinical severity, we analyzed the correlation between the serum NfL level and the following: trinucleotide repeat number, age at disease onset, disease duration, SARA score, and the cerebellar volume (Fig. 2). We also conducted subgroup analysis of the trinucleotide repeat number within SCA 2, 3, and 6, the three most common types of AD SCA (Supplementary Fig. S1).

The trinucleotide repeat number and serum NfL level were positively correlated (r = 0.47, *p*-value = 0.001) (Fig. 2a). Disease duration was positively correlated with the serum NfL level (r = 0.35, *p*-value = 0.015) (Fig. 2b). But disease duration/age, which reflects disease duration and onset, were not significantly correlated (r = 0.22, *p*-value = 0.136) (Fig. 2c). However, the disease duration/age × trinucleotide repeat number, which may reflect disease duration, onset, and clinical severity, were positively correlated with the serum NfL level (r = 0.33, *p*-value = 0.021) (Fig. 2d).

In addition, the SARA score and serum NfL were positively correlated (n = 33; r = 0.37, *p*-value = 0.033) (Fig. 2e), but the percentage of cerebellar volume within the TIV was not significantly correlated with the serum NfL level (n = 14; r = 0.02, *p*-value = 0.957 (Fig. 2f).

Among each of the most common SCAs, the trinucleotide repeat number and serum NfL level were not significantly correlated for SCA 2 (n = 19; r = 0.12, *p*-value = 0.633), SCA 3 (n = 13; r = − 0.20, *p*-value = 0.519), or SCA 6 (n = 9; r = − 0.48, *p*-value = 0.189) (Supplementary Fig. S1). Disease duration/age × trinucleotide repeat number also did not reveal a positive correlation with the serum NfL level for SCA 2 (n = 19; r = 0.04, *p*-value = 0.866), SCA 3 (n = 13; r = 0.25, *p*-value = 0.417) or SCA 6 (n = 9; r = − 0.63, *p*-value = 0.071).

## Discussion

Our most salient finding is that the serum NfL level is elevated in AD SCA patients and is correlated with the clinical severity markers of AD SCA such as trinucleotide repeat number, disease duration, disease duration/age × trinucleotide repeat number, and clinical severity (SARA score). Since disease severity and the rate of progression in AD SCA are quite heterogeneous among different types and individuals^1,2^, a reliable and easily measurable biomarker could be helpful for monitoring disease progression, evaluating treatment responses, and predicting prognosis^1,4,15^. In this regard, we suggest that serum NfL could serve as a potential biomarker for clinical severity in AD SCA.

Given that recent studies have demonstrated that serum NfL is correlated with clinical severity in SCA 3 patients^15,16^, in this study, the serum NfL level correlated well with various markers for clinical severity and the rate of disease progression in AD SCA. Serum NfL has been recognized as a promising biomarker for many neurodegenerative illnesses^4,10–15^. NfL reflects the neuronal damage in neurodegenerative process, considering previous studies demonstrated that serum NfL is related to neuronal damage. It is especially sensitive to axonal damage in long fiber tracts, such as spinocerebellar or corticospinal tracts^4,14,17^. In addition, the serum NfL level is easy to measure and follow up in series to detect disease progression. In our study, the serum NfL level was correlated with the trinucleotide repeat number, disease duration, disease duration/age × trinucleotide repeat number, and clinical severity. In addition, the serum NfL level alone was quite sensitive and specific for distinguishing groups with high disease severity from the mild group. These findings imply the serum NfL level’s broad clinical utility as a clinical biomarker of AD SCA.

Serum NfL could be used as progression marker and screening test for patients in the preclinical stage or with a genetic predisposition to AD SCA. Each type of AD SCA has hallmark non-ataxia symptoms such as parkinsonism, dementia, and chorea, except for SCA 6, which presents as pure ataxia^1,2,18^. Since several trials are currently ongoing to develop a pharmacological treatment for SCA^1,19–21^, screening for AD SCA using serum NfL could help to initiate disease-modifying treatment earlier and improve long-term outcomes. After further research of serum NfL in AD SCA including longitudinal studies with large cohort, it may be employed to evaluate treatment efficiency in developing disease-modifying therapies for SCA or to monitor long-term disease progression.

However, cerebellar atrophy is not correlated with serum NfL level. In a previous study, the correlation with cerebellar atrophy and clinical severity varied among different types of SCA^6^. Multiple factors (including SCA type, age, and sex) could affect the negative results of the relationship between serum NfL and the degree of cerebellar atrophy. Moreover, because serum NfL reflects ongoing neurodegeneration, it might not increase in the later stages of progressed cerebellar atrophy in AD SCA.

Our study has several limitations. First, we did not include patients in the preclinical stage. Longitudinal prospective research (including preclinical stage patients, as well as a comparison with other disorders of cerebellar ataxia) should be carried out to investigate whether serum NfL could serve as a screening test to promote early diagnosis. Second, the overall size of the cohort was too small to perform validation and subgroup analysis. In AUC analysis, to differentiate moderate and severe AD SCA from mild group by using serum NfL, validation with dividing cohort into training and test cohort is necessary to prevent overfitting. However, the sample size in AUC analysis was too small (n = 33) to perform validation. Also, the subgroup analysis of each severity group (11 patients each), and different types of AD SCA (SCA 2, 3, and 6) was too small (n = 19, 13, and 9 each) to analyze the correlation. Further, the number of patients who had brain MRI scans done within one year from the NfL sample collection was too small (n = 14). The analysis of correlation between cerebellar atrophy and serum NfL may be limited due to the sample size. More prospective studies of large AD SCA cohorts, with clinical severity evaluation and brain imaging, should be conducted. In addition, the size of the pathogenic expanded allele is different depending on different SCA types. So, analyzing the correlation between trinucleotide repeat number and serum NfL in AD SCA as a whole group could be limited. Last, our control group did not consist of completely healthy individuals. Several studies indicate that autoinflammation is linked to the pathomechanism of OH and POTS^22,23^; these inflammatory processes could also increase the serum NfL level. Notwithstanding, no previous studies have thus far shown direct neuronal damage to the central nervous system, which increases the serum NfL level and occurs in OH or POTS.

In conclusion, this study suggests serum NfL could be a promising biomarker of the neurodegenerative process and disease progression in AD SCA as a pilot study. Since serum NfL is easily measurable, it will be useful to monitor disease progression and treatment response more objectively. More research with longitudinal large, multicenter cohorts of SCA patients (including in the preclinical stage) with validation is warranted to further validate serum NfL as a rational biomarker for AD SCA.

## Methods

### Ethics declarations

We received approval for the study from Seoul National University Hospital (SNUH)’s Institutional Review Board. We carried out all methods in accordance with principles of the Declaration of Helsinki. We obtained written informed consent from all participants and sufficiently anonymized their information.

### Patient enrollment

We reviewed patients diagnosed with AD SCA who enrolled in the clinical trial “Effect of Nilotinib in Cerebellar Ataxia Patients” (NCT03932669) and visited SNUH’s neurology outpatient clinic between May and August of 2019. All patients were diagnosed with AD SCA through clinical evidence of progressive ataxia without any other acquired cause; this was confirmed by genetic testing, along with abnormal trinucleotide repeat expansions^1,2^. None of the patients had any other neurodegenerative or inflammatory illness except for SCA that could also affect the serum NfL level^10–14^. Genetic testing was performed via an AD SCA gene panel study combined with single-gene tests, including highly prevalent genotypes in South Korea (SCA types 1, 2, 3, 6, 7, 17, and DRPLA)^24^.

### Clinical evaluation

We gathered demographic data, as well as information on disease onset, disease period, brain MRI scans, serum NfL level, and genetic tests, by reviewing the participants’ medical records. At the participants’ first visit, four trained neurologists (H-R.S., EY.K., W-J.L., and HS.L.) determined clinical severity by using the SARA^5^. We evenly divided the AD SCA patients, whom we evaluated using the SARA (33 patients), into three subgroups: mild (SARA score ≤ 10; n = 11), moderate (10 < SARA score ≤ 13; n = 11), and severe (SARA score > 13; n = 11).

### Cerebellar volume analysis

We also analyzed the brain MRI scans of patients who underwent MRI within one year following the NfL sample collection. We obtained the cerebellar volume (CV) and total intracranial volume (TIV) from whole brain T1-weighted images using the Medical Image Processing, Analysis, and Visualization (MIPAV) software package^25^. The cerebellar volume was presented as the percentage of TIV (CV/TICV × 100) to adjust for the effect of TIV.

### Serum neurofilament light chain measurements

We obtained serum samples at the outpatient clinic with informed consent and stored them at − 80 °C in SST tubes^10,14,26^. The quantification of NfL was based on electrochemiluminescence (ECL) immunoassay. We used the capture monoclonal antibody (mAB) 47:3 (UmanDiagnostics, Umea, Sweden) and the Biotinylated detector mAB 2:1 (UmanDiagnostics, Umea, Sweden), and we employed MSD SULFO-TAG™ labeled streptavidin (MSD, Gaithersburg, MD) as a detection reagent to generate ECL^10,14,26^.

We added mAB 47:3 to coat the plates, then added 3% BSA in TBS per well as a blocking buffer^10,26^. All samples were evenly distributed on 96-well plates (Multi-Array plates; Meso Scale Discovery [MSD], Gaithersburg, MD)^14,26^. After adding mAB 2:1 as a detecting antibody and MSD SULFO-TAG to each well, we detected ECL signal using photodetectors (MSD SECTOR S 600, SECTOR Imager 2400 or QuickPlexSQ 120)^26^.

### Comparison to the control group

We also included 37 controls from an orthostatic intolerance cohort. We selected the controls from orthostatic intolerance patients who did not have any neurodegenerative, inflammatory, or systemic autoimmune disease that could also affect the serum NfL^10–14^. Among the controls, 29 (78.4%) had postural orthostatic tachycardia syndrome (POTS) and 8 (21.6%) had orthostatic hypotension (OH).

To compare the serum NfL levels of AD SCA patients with those of the healthy population, we also compared the serum NfL levels of AD SCA patients with healthy controls from two prior studies. One compared the serum NfL levels of ALS patients with healthy controls^14^, while the other compared the serum NfL levels of patients with various neurodegenerative diseases (including AD, ALS, and Guillain–Barre syndrome) to those of healthy controls^10^. Both studies used the same technique of quantifying serum NfL, which is based on ECL immunoassay^10,14,26^.

### Statistical analysis

The results are presented as the median [interquartile range (IQR)] or number (%). We performed an independent t-test to compare the age and serum NfL levels between the controls and the AD SCA patients, as well as a chi-square test to compare the sex distribution.

We carried out an analysis of variance (ANOVA) to compare the serum NfL levels between the controls and different severity groups of SCA patients, as well as an analysis of covariance (ANCOVA) to adjust for age. We used Pearson’s correlation coefficient to evaluate the correlation between serum NfL level and clinical severity among the AD SCA patients. In addition, we employed a receiver operating characteristic (ROC) curve for sensitivity analysis of the NfL cutoff values to distinguish the mild group from the moderate and severe groups. Further, we assessed the overall sensitivity and specificity as the area under the curve (AUC). We used SPSS version 25 (IBM, Armonk, NY) for all statistical analyses, and we considered two-tailed *p*-values < 0.05 to be statistically significant.

## Supplementary Information

Supplementary Figure S1.

Supplementary Tables.
