# Supplementary figures and images for: Serum neurofilament light chain as a severity marker for spinocerebellar ataxia
Source: Sci Rep. 2021 Jun 29;11:13517. doi: 10.1038/s41598-021-92855-z (PMC8241827; doi:10.1038/s41598-021-92855-z)

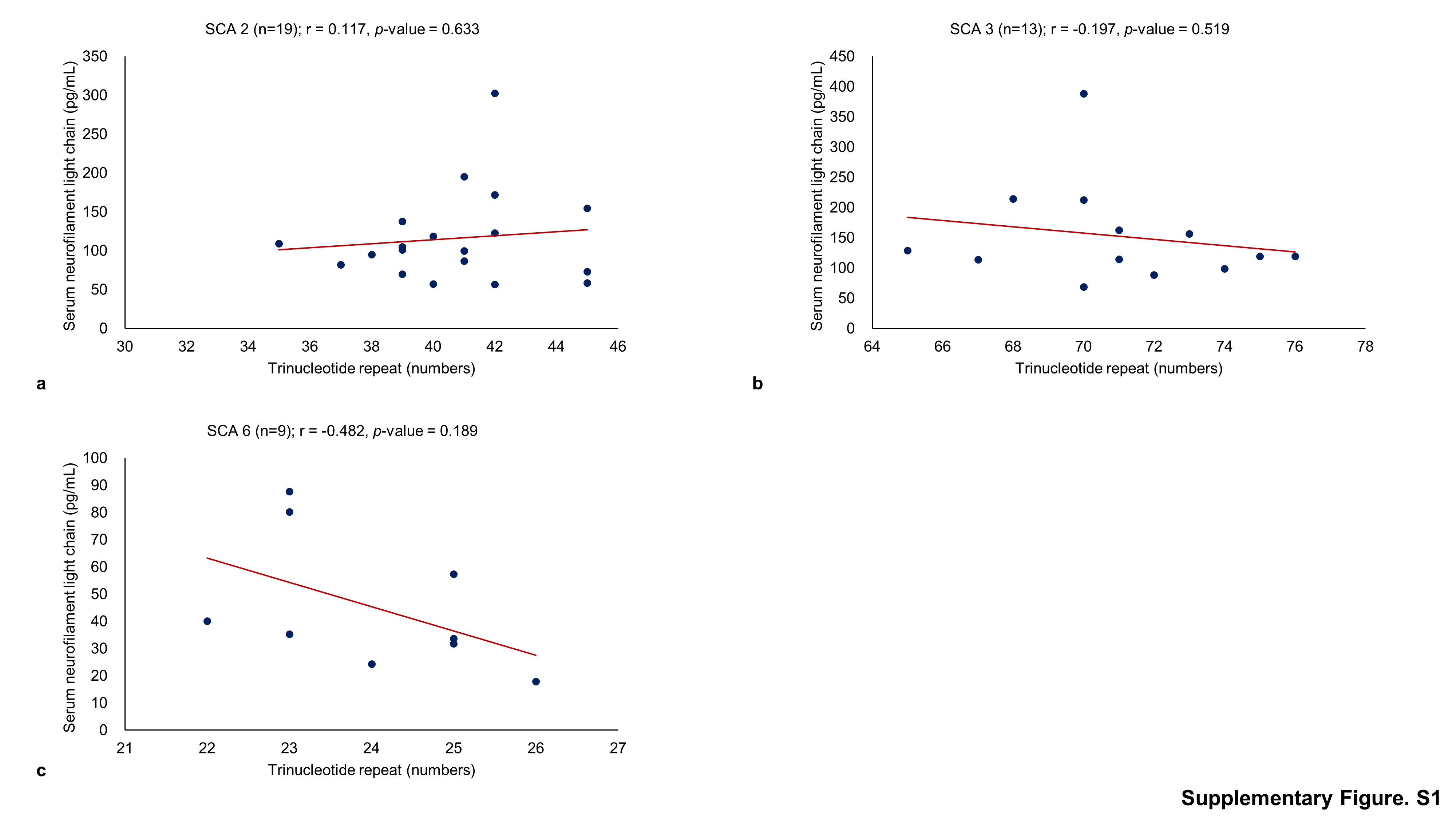

Supplement: Supplementary file 1 — Supplementary Figure S1. [file 41598_2021_92855_MOESM1_ESM.tif]
