# Supplementary material for: Serum neurofilament light chain as a severity marker for spinocerebellar ataxia
Source: Sci Rep. 2021 Jun 29;11:13517. doi: 10.1038/s41598-021-92855-z (PMC8241827; doi:10.1038/s41598-021-92855-z)
Supplement: Supplementary file 2 — Supplementary Tables. [file 41598_2021_92855_MOESM2_ESM.doc]

**Serum Neurofilament Light Chain as a Severity Marker for Spinocerebellar Ataxia**

Hye-Rim Shin^1^, Jangsup Moon^2,3,4^, Woo-Jin Lee^2,3,5^, Han Sang Lee^2,3,5^, Eun Young Kim^6^, Seoyi Shin^2,3^, Soon-Tae Lee^2,3^, Keun-Hwa Jung^2,3^, Kyung-Il Park^2,3,7^, Ki-Young Jung^2^, Sang Kun Lee^2,3^, Kon Chu^2,3*^

**Author affiliations**:

^1^Department of Neurology, Dankook University Hospital, Cheonan, Chungnam, South Korea

^2^Department of Neurology, Seoul National University Hospital, Seoul, South Korea

^3^Laboratory for Neurotherapeutics, Center for Medical Innovations, Biomedical Research Institute, Seoul National University Hospital, Seoul, South Korea

^4^Rare Disease Center, Seoul National University Hospital, Seoul, South Korea

^5^Center for Hospital Medicine, Seoul National University Hospital, Seoul, South Korea

^6^Department of Neurology, Chungnam National University Sejong Hospital, Sejong, South Korea

^7^Department of Neurology, Seoul National University Healthcare System Gangnam Center, Seoul, South Korea

**Supplementary Table S1. Demographics of autosomal-dominant spinocerebellar ataxia patients and serum neurofilament light chain levels on various types**.

| **Subject characteristics** | **SCA 1 (n=2)** | **SCA 2 (n=19)** | **SCA 3 (n=13)** | **SCA 6 (n=9)** | **SCA 7 (n=4)** | **SCA 17 (n=2)** |
| --- | --- | --- | --- | --- | --- | --- |
| Age – yr | 47.5 (45.3–50.0) | 36.0 (26.5–47.0) | 47.0 (36.0–57.0) | 47.0 (39.0–49.0) | 35.5 (22.5–53.0) | 45.5 (43.8–47.3) |
| Female sex – no. (%) | 2 (100) | 10 (52.6) | 10 (76.9) | 6 (66.7) | 3 (75.0) | 0 (0) |
| Trinucleotide repeat number | 46.5 (46.3–46.8) | 41.0 (39.0–42.0) | 71.0 (70.0–73.0) | 24.0 (23.0–25.0) | 48.5 (43.0–52.3) | 52.0 (46.5–57.5) |
| Disease onset – yr | 44.0 (41.5–46.5) | 29.0 (25.0–40.5) | 38.0 (31.0–48.0) | 44.0 (38.0–46.0) | 27.0 (21.0–38.8) | 44.0 (42.0–46.0) |
| Disease duration – yr | 3.5 (3.3–3.8) | 5.0 (2.0–7.0) | 6.0 (5.0–9.0) | 3.0 (2.0–4.0) | 7.0 (1.5–12.8) | 1.5 (1.3–1.8) |
| SARA (n = 33) | 32 (n=1) | 11.5 (10.0–15.0) (n=15) | 10.5 (9.0–12.9) (n=8) | 10.5 (9.0–12.0) (n=5) | 18.5 (14.8–22.3) (n=2) | 10.8 (9.6–11.9) (n=2) |
| Serum NfL (pg/mL) | 156.0 (140.3–171.7) | 101.3 (77.7–130.4) | 119.5 (114.1–162.9) | 35.3 (31.9–57.3) | 174.0 (163.8–185.0) | 87.4 (63.0–111.7) |

* Data are reported as the number (percentage), or as the median (interquartile range, IQR). SCA: spinocerebellar ataxia; SARA: Scale for the Assessment and Rating of Ataxia; NfL: = neurofilament light chain

**Supplementary Table S2. Patients characteristics of autosomal dominant spinocerebellar ataxia.**

|  | **Age/Sex** | **SCA type** | **Disease onset (yr)** | **Baseline SARA scale** | **Trinucleotide repeat number** | **Serum NfL (pg/mL)** |
| --- | --- | --- | --- | --- | --- | --- |
| **#1** | M/49 | SCA17 | 48 | 13 | 63 | 136.09 |
| **#2** | M/44 | SCA3 | 38 | 9 | 72 | 88.66 |
| **#3** | M/19 | SCA2 | 18 | 13 | 42 | 122.69 |
| **#4** | F/36 | SCA2 | 29 | Not examined | 41 | 86.61 |
| **#5** | F/49 | SCA6 | 46 | Not examined | 25 | 57.34 |
| **#6** | M/42 | SCA17 | 40 | 8.5 | 41 | 38.66 |
| **#7** | M/20 | SCA2 | 14 | 10 | 45 | 58.54 |
| **#8** | M/22 | SCA2 | 20 | 15 | 45 | 154.86 |
| **#9** | F/48 | SCA2 | 41 | 15.5 | 39 | 101.34 |
| **#10** | F/27 | SCA2 | 25 | 10 | 45 | 73.23 |
| **#11** | F/50 | SCA6 | 44 | Not examined | 22 | 40.15 |
| **#12** | F/52 | SCA1 | 49 | 32 | 46 | 187.44 |
| **#13** | F/57 | SCA3 | 48 | Not examined | 68 | 214.80 |
| **#14** | F/35 | SCA2 | 28 | 11 | 39 | 138.06 |
| **#15** | F/26 | SCA2 | 25 | Not examined | 40 | 118.82 |
| **#16** | F/71 | SCA7 | 59 | Not examined | 37 | 137.77 |
| **#17** | F/40 | SCA3 | 38 | Not examined | 71 | 162.94 |
| **#18** | F/18 | SCA7 | 18 | 11 | 53 | 213.31 |
| **#19** | M/33 | SCA2 | 26 | Not examined | 39 | 70.07 |
| **#20** | F/47 | SCA6 | 44 | 9 | 25 | 31.88 |
| **#21** | M/46 | SCA2 | 27 | 32 | 42 | 172.21 |
| **#22** | F/47 | SCA7 | 32 | 26 | 45 | 172.51 |
| **#23** | M/39 | SCA6 | 35 | 12 | 26 | 17.87 |
| **#24** | F/77 | SCA2 | 72 | 15 | 37 | 82.15 |
| **#25** | M/38 | SCA2 | 30 | Not examined | 41 | 99.83 |
| **#26** | F/58 | SCA3 | 37 | Not examined | 70 | 388.52 |
| **#27** | M/34 | SCA2 | 32 | 13.5 | 41 | 195.20 |
| **#28** | F/39 | SCA6 | 38 | Not examined | 25 | 33.73 |
| **#29** | F/56 | SCA3 | 52 | Not examined | 70 | 212.48 |
| **#30** | F/43 | SCA1 | 39 | Not examined | 47 | 124.54 |
| **#31** | F/27 | SCA3 | 14 | 20.5 | 74 | 99.07 |
| **#32** | M/45 | SCA6 | 38 | 14 | 23 | 35.32 |
| **#33** | F/50 | SCA3 | 45 | 17 | 71 | 114.51 |
| **#34** | F/58 | SCA3 | 50 | 11.5 | 67 | 114.14 |
| **#35** | F/49 | SCA6 | 47 | 10.5 | 23 | 80.29 |
| **#36** | F/46 | SCA2 | 38 | 11 | 42 | 302.86 |
| **#37** | F/58 | SCA6 | 57 | Not examined | 23 | 87.73 |
| **#38** | F/61 | SCA2 | 56 | 11.5 | 38 | 95.17 |
| **#39** | F/62 | SCA2 | 40 | 28 | 39 | 105.02 |
| **#40** | M/32 | SCA3 | 26 | Not examined | 75 | 119.16 |
| **#41** | M/36 | SCA3 | 31 | 10.5 | 73 | 156.74 |
| **#42** | F/47 | SCA3 | 41 | 8.5 | 70 | 69.14 |
| **#43** | M/50 | SCA2 | 48 | 6 | 35 | 109.49 |
| **#44** | M/24 | SCA7 | 22 | Not examined | 52 | 175.53 |
| **#45** | F/44 | SCA2 | 41 | 3.5 | 42 | 56.69 |
| **#46** | M/34 | SCA6 | 31 | 7.5 | 24 | 24.36 |
| **#47** | M/25 | SCA2 | 20 | 6.5 | 40 | 57.43 |
| **#48** | F/26 | SCA3 | 25 | 10.5 | 76 | 119.53 |
| **#49** | F/72 | SCA3 | 60 | 9 | 65 | 129.02 |

* SCA = spinocerebellar ataxia; SARA = Scale for the Assessment and Rating of Ataxia; NfL = neurofilament light chain

* In patients who were not evaluated baseline SARA scale at their first visit, baseline SARA scale is presented as ‘Not examined’.

**Supplementary Table S3**. Serum neurofilament level in controls and spinocerebellar ataxia patients.

|  | **Total** | **Median (pg/mL)** | **Range (pg/mL)** |
| --- | --- | --- | --- |
| **Healthy Controls in Benatar et al, 2018 (compared with ALS)** | 34 | 10.7 | 0.4–33.5 |
| **Healthy Controls in Gaiottino et al, 2013**  **(compared with neurodegenerative diseases)** | 67 | 3.3 | 2.0–5.4 |
| **AD SCA in current study** | 49 | 109.5 | 17.9–388.5 |

* SCA = spinocerebellar ataxia; ALS = amyotrophic lateral sclerosis.
